# Honor to Our Brother

**Published:** 1896-05

**Authors:** 


### HONOR TO OUR BROTHER.

The Board of Freeholders of New Brunswick, N. J., has elected Dr. Samuel Long, County Physician, to succeed Dr. Carroll, of the old school, the former incumbent.

*The Daily Home News*, of New Brunswick, in speaking of it says editorially :

" It is quite a feather in the cap of the practicing homœopathists of this community that one of their own has been recognized as a desirable person for the office of County Physician. Thus it is seen that the selection of Dr. Samuel Long by the Board of Freeholders yesterday over Dr. Carroll had a peculiar significance, for it is probably the first time in the history of the county that a new school disciple has been thus recognized. The vote was 10 to 9, and the Republicans voted as a unit for Dr. Long, although there had been no caucus. And we have no doubt that Dr. Long's politics were unknown to the members of the Board, as they are this minute to us."

A correspondent of the same journal writes as follows :

" HOMŒOPATHY GOT A BOOST.—How certain esteemed physicians of this city would have jumped into the fight had they supposed for a moment that the Board of Freeholders intended to elect a homœopathic physician as County Physician. We should have seen some wire-pulling that would have been intensely interesting. As it is we can congratulate Dr. Long. The foolish prejudice against the new school has received a body blow.—REMUS."

THE HOMŒOPATHIC PHYSICIAN congratulates New Brunswick upon its selection of so able a physician as Dr. Long, who is a homœopathist, not only in name, but most strict in his practice of the scientific doctrines of Hahnemann.

